# Crosstalk Between Antioxidants and Adipogenesis: Mechanistic Pathways and Their Roles in Metabolic Health

**DOI:** 10.3390/antiox14020203

**Published:** 2025-02-10

**Authors:** Minghao Fu, Kyung-Sik Yoon, Joohun Ha, Insug Kang, Wonchae Choe

**Affiliations:** 1 Department of Biochemistry and Molecular Biology, School of Medicine, Kyung Hee University, Seoul 02447, Republic of Korea; andrewfupg@khu.ac.kr (M.F.); sky9999@khu.ac.kr (K.-S.Y.); hajh@khu.ac.kr (J.H.); iskang@khu.ac.kr (I.K.); 2 Department of Biomedical Science, Graduate School, Kyung Hee University, Seoul 02447, Republic of Korea

**Keywords:** oxidative stress, adipogenesis, PPARγ, C/EBPα, natural antioxidants, epigenetic mechanisms, insulin resistance, obesity, personalized antioxidant therapies, endogenous antioxidants

## Abstract

The interplay between oxidative stress and adipogenesis is a critical factor in the development of obesity and its associated metabolic disorders. Excessive reactive oxygen species (ROS) disrupt key transcription factors such as peroxisome proliferator-activated receptor gamma (PPARγ) and CCAAT/enhancer-binding protein alpha (C/EBPα), impairing lipid metabolism, promoting adipocyte dysfunction, and exacerbating inflammation and insulin resistance. Antioxidants, classified as endogenous (e.g., glutathione, superoxide dismutase, and catalase) and exogenous (e.g., polyphenols, flavonoids, and vitamins C and E), are pivotal in mitigating these effects by restoring redox balance and preserving adipocyte functionality. Endogenous antioxidants neutralize ROS and safeguard cellular structures; however, under heightened oxidative stress, these defenses are often insufficient, necessitating dietary supplementation. Exogenous antioxidants derived from plant-based sources, such as polyphenols and vitamins, act through direct ROS scavenging, upregulation of endogenous antioxidant enzymes, and modulation of key signaling pathways like nuclear factor kappa B (NF-κB) and PPARγ, reducing lipid peroxidation, inflammation, and adipocyte dysfunction. Furthermore, they influence epigenetic regulation and transcriptional networks to restore adipocyte differentiation and limit lipid accumulation. Antioxidant-rich diets, including the Mediterranean diet, are strongly associated with improved metabolic health, reduced obesity rates, and enhanced insulin sensitivity. Advances in personalized antioxidant therapies, guided by biomarkers of oxidative stress and supported by novel delivery systems, present promising avenues for optimizing therapeutic interventions. This review, “Crosstalk Between Antioxidants and Adipogenesis: Mechanistic Pathways and Their Role in Metabolic Health”, highlights the mechanistic pathways by which antioxidants regulate oxidative stress and adipogenesis to enhance metabolic health.

## 1. Introduction

Obesity has surged globally, affecting diverse populations across socioeconomic and geographic boundaries far beyond its earlier association with wealthier nations [1]. As of today, more than 650 million adults are classified as obese, and this number continues to grow at an alarming rate. This growing public health crisis is exacerbating healthcare systems and increasing the prevalence of non-communicable diseases such as diabetes, hypertension, and cardiovascular diseases [2]. Adipogenesis is the process by which mesenchymal stem cells differentiate into adipocytes, a process that is regulated by transcription factors such as PPARγ and C/EBPα [3,4]. Mature adipocytes are the primary cells responsible for the storage of fat in the form of triglycerides. The process of lipogenesis plays a pivotal role in energy storage during periods of calorie surplus [5]. However, in cases of obesity, an excessive amount of lipogenesis generates elevated levels of ROS as metabolic byproducts, which disrupts the cellular redox balance and contributes to oxidative stress [6]. Elevated ROS levels have been shown to impair the function of critical transcription factors, such as PPARγ and C/EBPα, which play a pivotal role in regulating lipid metabolism. This, in turn, results in the disruption of adipocyte differentiation and the subsequent impairment of their function [7]. These effects extend to damage to cellular membranes, proteins, and DNA, further compounding metabolic dysfunction. Low-grade inflammation, driven by macrophage infiltration into adipose tissue, further exacerbates oxidative stress [8,9]. This inflammatory environment is characterized by the secretion of cytokines, including tumor necrosis factor-alpha (TNF-α), interleukin-6 (IL-6), and interferon-gamma (IFN-γ) [10]. The role of IFN-γ in this process is particularly noteworthy, as it serves to amplify the activation of macrophages and the inflammatory signaling that ensues, thereby perpetuating the cycle of oxidative stress and inflammation [11]. These cytokines subsequently activate NF-κB, thereby perpetuating the pro-inflammatory milieu that ultimately compromises adipocyte function [12,13,14]. This results in disturbances in lipid metabolism, which in turn exacerbates the microenvironment of adipose tissue by activating pro-inflammatory pathways. The utilization of antioxidants has emerged as a pivotal strategy to disrupt the cycle of oxidative damage and restore cellular homeostasis; antioxidants counteract ROS-induced damage by neutralizing free radicals, modulating lipogenesis-associated signaling pathways, and preserving transcription factors like PPARγ and C/EBPα, which are essential for adipocyte differentiation and function [15]. Consequently, the research community has pivoted towards investigating the potential of antioxidants in enhancing metabolic well-being [16,17,18]. A comprehensive understanding of the mechanisms by which antioxidants modulate lipid metabolism and reduce oxidative stress is essential for fully appreciating their potential therapeutic value in the context of obesity and related metabolic disorders [19,20]. The preservation of adipocyte function is imperative in the prevention of excessive lipid accumulation and the subsequent development of obesity [21]. While the protective role of antioxidants against oxidative stress has been extensively documented, particularly in relation to fat cell health, less attention has been given to the dynamic interplay between endogenous antioxidants and those derived from natural sources. Endogenous antioxidants, including glutathione and enzymes such as SOD and catalase, play a critical role in managing oxidative stress. However, in cases of obesity or suboptimal dietary conditions, an increase in ROS production can overwhelm these defenses, leaving cells susceptible to oxidative damage [22]. In addition to the body’s intrinsic antioxidant defenses, the inclusion of dietary antioxidants derived from plant-based foods provides a complementary approach to combating oxidative stress [23,24]. Compounds such as polyphenols, vitamins C and E, and carotenoids have demonstrated the capacity to bolster cellular defenses and enhance adipocyte functionality under conditions of obesity [19,25].

This article will examine the specific links and relationships between antioxidants and adipogenesis, with a particular focus on the effects of natural antioxidants on this process.

## 2. Mechanisms of Adipogenesis

This section explores the biological mechanisms underlying adipocyte differentiation, the role of key transcription factors, and the influence of oxidative stress and environmental factors on adipogenesis. Adipogenesis is a tightly regulated, multi-step process that begins with MSC commitment to the adipogenic lineage and culminates in the maturation of fully differentiated adipocytes [26,27]. This process is influenced by transcriptional, epigenetic, and environmental factors, which become particularly significant under pathological conditions such as obesity [28,29].

### 2.1. Adipocyte Physiology

Adipocytes, the key energy storage and endocrine cells of adipose tissue are classified into three types based on localization and function: white adipose tissue (WAT), brown adipose tissue (BAT), and beige adipocytes [30,31].

#### 2.1.1. White Adipose Tissue (WAT)

WAT plays a pivotal role in energy storage, predominantly in the form of triglycerides. This adipose tissue is further categorized into subcutaneous adipose tissue (SAT) and visceral adipose tissue (VAT) [32,33]. SAT, located beneath the skin, plays a central role in energy homeostasis. In contrast, VAT surrounds internal organs and is closely associated with metabolic dysfunction when in excess [34].

#### 2.1.2. Brown Adipose Tissue (BAT)

BAT dissipates energy as heat through uncoupling protein 1 (UCP1)-mediated thermogenesis. Rich in mitochondria, BAT counters energy surplus and is activated by cold exposure or caloric excess [35].

#### 2.1.3. Beige Adipocytes

Beige adipocytes form within WAT during “browning” induced by cold exposure or adrenergic signaling. These cells exhibit BAT-like thermogenic properties, enhancing energy expenditure and protecting against obesity [36].

### 2.2. Commitment and Epigenetic Modulation

The commitment of MSCs to the adipogenic lineage marks the first stage of adipogenesis and is orchestrated by key transcription factors, particularly peroxisome PPARγ and C/EBPα. These transcription factors activate downstream adipogenic genes, initiating the differentiation process and ensuring the precise maturation of functional adipocytes under normal conditions [3,37,38].

Recent research highlights the critical role of epigenetic modifications during early adipocyte differentiation. Modifications such as DNA methylation and histone acetylation regulate chromatin accessibility, determining the activation or suppression of adipogenic genes [39]. Environmental factors, including dietary habits and metabolic status, influence these epigenetic signatures. Sustained cues such as pro-inflammatory cytokines or high-fat diets during early development establish persistent epigenetic marks, predisposing MSCs to pathological adipogenesis in later stages [28,40]. Thus, adipogenesis is regulated not only by transcription factors but also by long-lasting epigenetic changes shaped by environmental conditions [40,41].

### 2.3. Environmental and Pathological Influences

Adipogenesis is profoundly influenced by environmental factors and pathological conditions, particularly in the context of obesity [42,43]. While transcriptional and epigenetic mechanisms establish the foundation for normal adipocyte differentiation, obesity introduces chronic low-grade inflammation, oxidative stress, and early-life environmental exposures that disrupt this tightly regulated process [44].

#### 2.3.1. Inflammatory Disruption

Pro-inflammatory cytokines, including TNF-α, IL-6, and IFN-γ, exhibit elevated levels in obesity cases and disrupt adipogenesis by impairing the function of transcriptional regulators such as PPARγ and C/EBPα [45,46,47]. This disruption skews adipocyte differentiation toward hypertrophy and dysfunctional lipid metabolism. Furthermore, the intensification of inflammatory signaling by IFN-γ fosters the perpetuation of a pro-inflammatory microenvironment, thereby contributing to the maintenance of adipocyte dysfunction [48,49,50].

#### 2.3.2. Oxidative Damage

Excessive ROS in obesity exacerbates adipogenic dysfunction by causing oxidative damage to cellular membranes, proteins, and DNA. ROS impair critical transcriptional regulators like PPARγ, further disrupting adipocyte differentiation and lipid regulation [51]. This oxidative imbalance creates a feedback loop, amplifying adipocyte dysfunction and metabolic instability.

#### 2.3.3. Early-Life Exposures

Early-life environmental factors, including nutrient excess and exposure to inflammatory stimuli, have been demonstrated to establish persistent epigenetic changes that predispose MSCs to enhanced adipogenic differentiation later in life [52,53]. These modifications, influenced by early dietary habits and metabolic conditions, create a lasting impact on cellular programming and link developmental exposures to an increased risk of obesity in adulthood [54,55].

Adipogenesis is a process that is subject to precise regulation by transcriptional and epigenetic mechanisms. Obesity has been demonstrated to disrupt these processes through the mechanisms of inflammation, oxidative stress, and environmental factors, resulting in metabolic dysfunction. This underscores the critical role of transcription factors in adipocyte regulation [56,57,58].

## 3. The Regulatory Role of Key Transcription Factors in Adipogenesis

The process of adipocyte differentiation is regulated by three key transcription factors: Together, PPARγ, C/EBPα, and Sterol SREBP-1c regulate the expression of genes involved in lipid metabolism, adipocyte development, and insulin sensitivity [59]. The roles of these factors are not only interdependent but also subject to dynamic influence from metabolic and environmental conditions.

Adipocyte differentiation is not only directly influenced by transcription factors such as PPARγ and SREBP-1c but also by upstream signaling cascades including insulin and mTOR [60]. These pathways integrate various metabolic signals including nutrient availability and oxidative stress to finely tune the transcriptional network essential for adipocyte differentiation [61,62].

**Figure 1:**
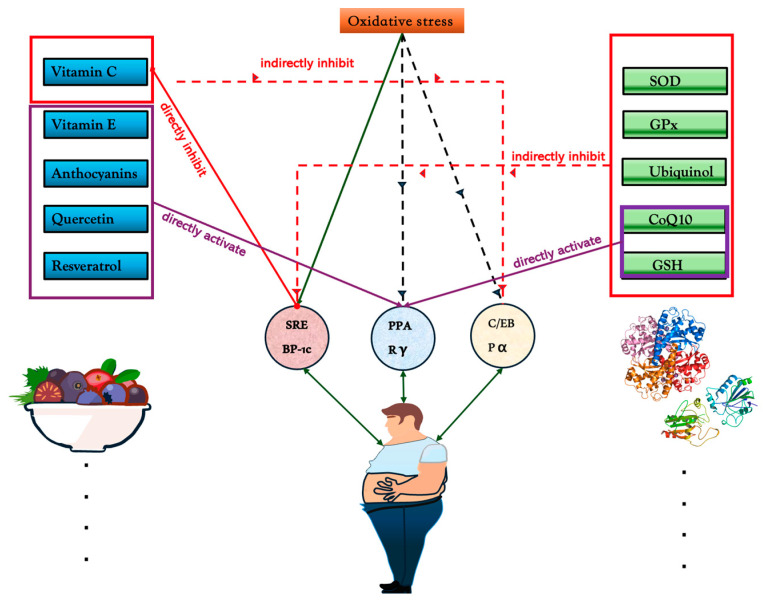
Mechanisms of antioxidant regulation on oxidative stress and transcription factors SREBP-1c, PPARγ, and C/EBPα. Natural antioxidants, including vitamin C, vitamin E, anthocyanins, quercetin, and resveratrol, directly or indirectly modulate these factors. Vitamin C and quercetin directly inhibit SREBP-1c, reducing lipid biosynthesis, while resveratrol activates PPARγ, enhancing anti-inflammatory responses. Endogenous antioxidants like SOD, GPx, ubiquinol, CoQ10, and GSH help maintain redox balance. CoQ10 and GSH directly activate PPARγ, while GPx and ubiquinol indirectly suppress SREBP-1c. Molecular structures of ubiquinone and ubiquinol demonstrate their roles in redox cycling, highlighting the essential function of antioxidants in metabolic regulation.

As shown in Figure 1, the differentiation of adipocytes is regulated by several key transcription factors, including PPAR γ, C/EBPα, and SREBP-1c, which collectively direct lipid metabolism and storage [63,64,65]. As the primary regulator, PPARγ is responsible for directing processes such as lipid uptake, triglyceride storage, and glucose metabolism [66,67]. In conjunction with PPARγ, C/EBPα enhances insulin sensitivity and facilitates adipocyte maturation by activating lipogenic genes. Meanwhile, SREBP-1c, which is responsive to both insulin and oxidative stress, plays a vital role in fatty acid synthesis, contributing to the accumulation of lipids within adipocytes [68,69]. The interaction among these transcription factors is of great importance for the maintenance of adipocyte function and metabolic homeostasis. Upstream signaling cascades, particularly the insulin-mTOR pathway, incorporate metabolic signals to regulate adipogenesis by establishing a positive feedback loop through PPARγ, which reinforces both adipocyte differentiation and lipid storage [60]. Nevertheless, prolonged SREBP-1c activation in obesity can result in excessive lipid synthesis, leading to the development of hypertrophic adipocytes and lipotoxicity, which ultimately impairs insulin sensitivity [70].

ROS functions as an additional regulatory layer, acting as signaling molecules at moderate levels but causing dysfunction when in excess [71]. This ROS-mediated pathway contributes to the development of abnormal adipocyte hypertrophy and excessive fat storage, which are key features of obesity [72,73]. Furthermore, epigenetic mechanisms, including histone methylation (e.g., H3K27me3) and non-coding RNAs (e.g., miR-27), provide additional dynamic control over the process of adipogenesis [74]. Histone methylation can suppress the expression of genes that direct alternative cell fates, thereby directing MSCs toward the adipocyte lineage [75,76]. MicroRNAs can also refine the process of adipogenesis by targeting transcription factors such as PPARγ, thus ensuring precise regulation [77]. In the context of obesity, chronic inflammation serves to exacerbate the dysfunction of adipocytes via the activation of NF-κB, which in turn induces the production of pro-inflammatory cytokines (e.g., TNF-α). These cytokines impair insulin signaling and destabilize C/EBPα and PPARγ, thereby further driving the proliferation of adipocytes and the development of insulin resistance [78,79]. Therapeutic strategies that target oxidative stress and inflammation, such as antioxidants (resveratrol and curcumin) and miRNA-based therapies, show promise in reducing lipid accumulation and improving insulin sensitivity [80,81]. These approaches offer targeted solutions for the management of obesity and related metabolic disorders.

## 4. Antioxidants: Types and Their Roles in Cellular Health

Natural antioxidants, predominantly sourced from dietary intake, serve as a fundamental line of defense against oxidative stress by mitigating ROS and upholding cellular integrity [82,83]. These exogenous antioxidants encompass polyphenols, vitamins C and E, and carotenoids, each with distinctive mechanisms of action that act in a synergistic manner to reinforce cellular antioxidant capacity [84,85]. Polyphenols, an extensive category of compounds found in a range of plant-based foods, including berries, green tea, and nuts, function not only as direct scavengers of ROS but also as modulators of redox-sensitive signaling pathways [86,87]. Polyphenols also bind to metal ions that catalyze oxidative reactions, thereby further impeding ROS formation at the cellular level [88]. Furthermore, they are known to induce endogenous antioxidant enzymes, such as SOD and catalase, through the upregulation of gene expression, thereby amplifying intrinsic antioxidant defenses [89]. Vitamin C (ascorbic acid), which is predominantly found in citrus fruits, bell peppers, and leafy greens, is a potent water-soluble antioxidant that operates in both intracellular and extracellular matrices [90,91]. Its primary function is to donate electrons in order to neutralize ROS, and it plays a pivotal role in regenerating other antioxidants, notably vitamin E. Vitamin E (α-tocopherol), a lipid-soluble antioxidant obtained from nuts, seeds, and vegetable oils, incorporates into cellular membranes, where it protects against lipid peroxidation, a process that can destabilize membrane integrity and functionality [92,93]. Carotenoids, such as beta-carotene and lycopene, which are prevalent in colorful fruits and vegetables, have been demonstrated to be particularly effective at quenching singlet oxygen, a highly reactive form of ROS [94,95]. This offers additional membrane stabilization. Collectively, these natural antioxidants contribute to a robust antioxidant defense system, thereby safeguarding cellular structures and biomolecular integrity against oxidative damage. This underscores the critical role of a diverse, antioxidant-rich diet in maintaining cellular health.

In addition to dietary antioxidants, the body synthesizes a variety of endogenous antioxidants that are essential for maintaining intracellular redox balance [96,97]. The endogenous antioxidant system is comprised of a number of enzymatic antioxidants, including SOD, catalase, glutathione peroxidase (GPx), and the thioredoxin system [98]. Each of these fulfills a specific role within the cellular antioxidant defense mechanisms. SOD catalyzes the conversion of superoxide radicals, highly reactive byproducts of mitochondrial respiration, into hydrogen peroxide [99]. This is subsequently converted into water and oxygen by catalase and GPx, respectively, thereby averting potential oxidative damage [100]. Catalase, which is predominantly localized within peroxisomes, exhibits remarkable catalytic efficiency in decomposing hydrogen peroxide and acts as a rapid-response antioxidant under conditions of high oxidative stress. GPx employs glutathione, a tripeptide with a cysteine residue, as a substrate to detoxify hydrogen peroxide and lipid peroxides, thereby protecting biomolecules such as lipids, proteins, and nucleic acids from oxidative modifications [101,102]. The thioredoxin system, comprising thioredoxin and thioredoxin reductase, facilitates the reduction of oxidized proteins and contributes to DNA synthesis and repair, cellular proliferation, and apoptosis regulation, thereby further underscoring its importance in maintaining cellular viability under oxidative conditions [103]. Collectively, these endogenous systems, in conjunction with dietary antioxidants, constitute an integrated network that dynamically regulates cellular redox states, thereby preventing the pathogenesis of oxidative stress-related diseases.

In addition to their primary function in the neutralization of ROS, both natural and endogenous antioxidants exhibit dual functionality by modulating inflammatory pathways, lipid metabolism, and adipogenesis, thereby contributing to systemic metabolic homeostasis [104,105]. By attenuating oxidative stress, antioxidants inhibit the activation of the pro-inflammatory transcription factor NF-κB, which in turn leads to a reduction in the expression of cytokines such as TNF-α [106,107,108]. These cytokines are implicated in chronic inflammation and metabolic dysfunctions. Moreover, antioxidants exert a protective effect on lipid metabolism by mitigating lipid peroxidation, which in turn prevents the aberrant accumulation of lipids and the hypertrophic expansion of adipocytes, which are commonly observed in obesity [109,110,111]. This regulatory influence extends to the process of adipogenesis, where antioxidants play a critical role in ensuring normal adipocyte differentiation and function, thus preventing metabolic derangements associated with excessive fat deposition [111,112,113]. Collectively, the multifaceted actions of antioxidants—ranging from ROS neutralization to inflammatory modulation and lipid regulation—demonstrate their indispensable role in cellular resilience and the maintenance of metabolic equilibrium [97,114]. Consequently, they are regarded as pivotal agents in the prevention of oxidative stress-related diseases and age-associated cellular decline

## 5. Interaction Between Antioxidants and Adipogenesis

Antioxidants exert their influence on adipogenesis through complex interactions with key molecular pathways that regulate adipocyte differentiation, particularly the NF-κB, PPARγ, and ROS signaling [115,116].These cytokines amplify pro-inflammatory signaling, creating a feedback loop that promotes adipocyte differentiation and excessive lipid storage. Additionally, elevated ROS levels further activate NF-κB, thereby fostering a highly pro-inflammatory environment that perpetuates adipogenesis [25]. In this context, antioxidants such as resveratrol, curcumin, and various polyphenols act by directly neutralizing ROS, thereby dampening NF-κB activation [81,117,118]. This reduction in cytokine production disrupts the pro-inflammatory cycle, weakening one of the key pathways that perpetuate adipogenesis. Meanwhile, PPARγ, a master transcription factor in adipocyte maturation, normally regulates lipid uptake and triglyceride storage [119]. Under oxidative conditions, excessive PPARγ activation promotes pathological lipid storage and adipocyte hypertrophy, contributing to metabolic dysfunction. Antioxidants assist in maintaining the regulatory equilibrium of PPARγ by reducing ROS levels, thereby ensuring that PPARγ activity remains within the optimal ranges that support physiological lipid storage [120,121]. Compounds such as epigallocatechin gallate (EGCG), a potent green tea polyphenol, exemplify this equilibrium by modulating PPARγ expression, ensuring healthy lipid metabolism without the pathological adipogenesis associated with metabolic dysfunction [122,123,124].

**Figure 2:**
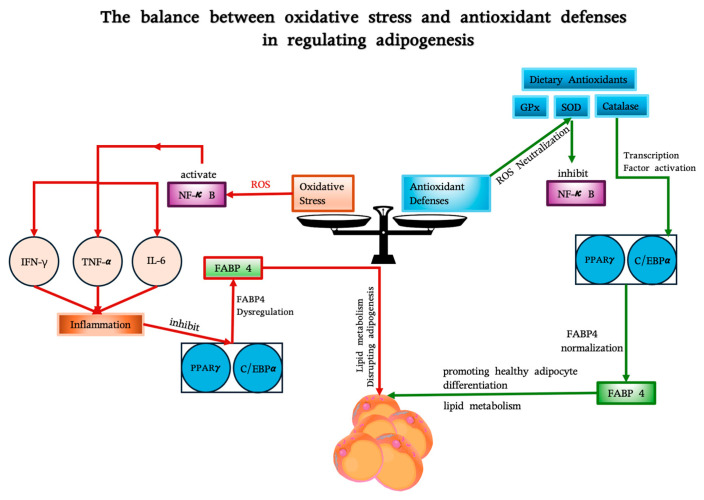
The balance between oxidative stress and antioxidant defenses in regulating FABP4 and adipogenesis. Oxidative stress (left), driven by ROS and inflammation (via NF-κB and cytokines like IFN-γ, TNF-α, and IL-6), suppresses transcription factors PPARγ and C/EBPα, leading to FABP4 dysregulation and impaired adipocyte function. Antioxidant defenses (right), including endogenous enzymes (GPx, SOD, catalase) and dietary antioxidants (e.g., resveratrol, curcumin), neutralize ROS, inhibit NF-κB, and restore transcription factor activity, normalizing FABP4 expression. FABP4 links these pathways, facilitating healthy adipogenesis by supporting lipid metabolism and adipocyte differentiation.

In addition to regulating these pathways, antioxidants exert a direct influence on adipocyte proliferation and the overall expansion of fat mass, which is essential for maintaining healthy adipose tissue [125,126,127]. Vitamins C and E serve as efficacious defenders against these effects, with vitamin E, in particular, integrating into adipocyte membranes to prevent lipid peroxidation, thereby preserving cellular structure and reducing the excessive proliferation associated with adiposity [93,128]. Conversely, polyphenols such as quercetin and EGCG have been observed to downregulate pivotal adipogenic genes implicated in lipid biosynthesis and cell cycle progression, thereby exerting a direct inhibitory effect on adipocyte proliferation [129]. By reducing oxidative stress at the cellular level, these antioxidants help to inhibit the pathological growth of adipose tissue, thereby demonstrating their therapeutic relevance in the management of obesity and the promotion of metabolic resilience. Figure 2 depicts the interplay between oxidative stress and antioxidant defenses in adipogenesis, with fatty acid binding protein 4 (FABP4) as a central regulator. FABP4 is one of the most abundant proteins in adipocytes and plays an important role in maintaining adipocyte homeostasis by regulating lipolysis and lipogenesis [130,131,132].

Furthermore, the anti-inflammatory properties of antioxidants influence their impact on adipogenesis, particularly through their capacity to disrupt the production of pro-inflammatory cytokines, which drive abnormal adipocyte expansion. Chronic inflammation, which is particularly prevalent in obesity, generates a continuous influx of cytokines, such as TNF-α and IL-6, that maintain a self-reinforcing cycle of NF-κB activation and oxidative stress [133,134]. The pro-inflammatory environment has been demonstrated to have a deleterious effect on adipocyte proliferation, while concomitantly disrupting systemic metabolic stability. Antioxidants such as resveratrol, curcumin, and carotenoids intervene by inhibiting NF-κB activation, thereby reducing cytokine levels and shifting the balance toward an anti-inflammatory state [119,135]. Some antioxidants even enhance anti-inflammatory cytokines like IL-10, thereby providing a dual approach that suppresses pro-inflammatory signals while stabilizing adipose tissue [136,137,138]. By alleviating inflammation and oxidative stress, antioxidants create a more resilient metabolic environment, thereby reducing the risk of insulin resistance and metabolic dysfunction, particularly in the context of obesity and its associated health risks. This combined anti-inflammatory and ROS-neutralizing effect demonstrates the versatility of antioxidants as agents capable of reshaping adipogenic pathways toward healthier, more balanced adipocyte function.

### 5.1. Antioxidants: Sources and Mechanisms

Antioxidants play a pivotal role in counteracting oxidative stress and inflammation, both of which are central to the development of metabolic dysfunction. Endogenous antioxidants, including SOD, catalase, and GPx, act as the primary line of defense by neutralizing ROS and maintaining cellular redox equilibrium [139,140]. These intrinsic systems are further supported by dietary antioxidants, which include polyphenols, carotenoids, and vitamins C and E, predominantly sourced from plant-based foods like fruits, vegetables, nuts, and seeds [141,142]. The function of these dietary antioxidants is twofold: they scavenge ROS and influence key molecular pathways, particularly those involving the transcription factors NF-κB and PPARγ. These transcription factors are critical for regulating adipocyte differentiation and lipid metabolism, and their dysfunction is closely linked to metabolic disorders [25,143]. Antioxidants aid in preserving the activity of these transcription factors, thereby ensuring that adipocytes maintain functional lipid storage and energy balance. By mitigating oxidative damage and restoring homeostasis in cellular signaling, antioxidants contribute to a reduction in the risk of pathological adipocyte hypertrophy, a hallmark of obesity [25,144,145].

### 5.2. Experimental Evidence and Relevant Research

Experimental studies have yielded valuable insights into the influence of antioxidants on adipose tissue health. In vitro investigations reveal that polyphenols such as resveratrol and EGCG reduce lipid accumulation in adipocytes by suppressing the expression of lipogenic enzymes and modulating inflammatory pathways [146,147]. Animal studies further demonstrate the effectiveness of antioxidant supplementation in improving insulin sensitivity, lowering oxidative damage, and alleviating chronic inflammation within adipose tissue [148,149]. These findings underscore the potential of antioxidants to enhance metabolic health by targeting the underlying mechanisms of adipocyte dysfunction. While clinical research has yielded more variable results, it continues to offer additional support for these benefits. Long-term consumption of antioxidant-rich diets has been associated with decreased oxidative stress markers and improved metabolic profiles in obese individuals [150]. However, the observed effects are often context-dependent, shaped by factors such as the dosage and bioavailability of antioxidants, as well as individual differences in metabolic states. This variability underscores the necessity of tailoring antioxidant interventions to meet specific physiological and metabolic needs [151,152,153].

Antioxidants have been demonstrated to play a vital role in protecting adipose tissue by reducing oxidative stress, regulating transcriptional activity, and controlling inflammation. The potential benefits of antioxidants as a therapeutic modality for obesity-related metabolic disorders are significant. However, the efficacy of antioxidants is contingent upon individual metabolic conditions, necessitating a delicate balance between oxidative stress and antioxidant defenses [154,155]. Consequently, further research is necessary to enhance and personalize antioxidant-based therapeutic interventions.

## 6. Role of Endogenous Antioxidants in Combating Obesity-Related Oxidative Stress

Endogenous antioxidants play a pivotal role in mitigating the effects of oxidative stress associated with obesity. They protect cellular structures, support metabolic balance, and prevent inflammatory responses. Table 1 highlights the roles and mechanisms of endogenous antioxidants in combating obesity-related oxidative stress, reinforcing the article’s focus on targeted strategies for improving metabolic health.

In conclusion, the aforementioned endogenous antioxidants—GSH, SOD, catalase, GPx, the thioredoxin system, CoQ10, HO-1, PRXs, PON1, and ubiquinol—function collectively to form a multi-layered defense system [170]. These antioxidants operate at various cellular sites, protecting adipocytes from oxidative stress, maintaining redox balance, and supporting metabolic function. Collectively, they mitigate the effects of ROS in obesity, reducing metabolic dysregulation and inflammation to safeguard cellular health [170,171].

## 7. Role of Natural Antioxidants in Adipogenesis and Oxidative Stress

The use of natural antioxidants derived from plant sources is of significant importance in the management of oxidative stress and the modulation of lipogenesis, both of which are pivotal in addressing metabolic disorders such as obesity [19,172]. Bioactive compounds, including polyphenols, flavonoids, carotenoids, and vitamins C and E, derived from fruits, vegetables, nuts, and seeds, confer multifaceted benefits by neutralizing ROS while augmenting endogenous antioxidant systems [85,173,174]. For example, tea polyphenols, particularly EGCG, act as dual agents, scavenging ROS and upregulating key antioxidant enzymes such as GPx and SOD [124,175]. This dual action helps to maintain cellular redox equilibrium in the lipid-rich and oxidative environment of adipose tissue. Furthermore, EGCG modulates adipogenesis by downregulating transcription factors such as PPARγ and C/EBPα, which curtails adipocyte maturation and limits lipid storage [15,119]. Moreover, evidence indicates that tea polyphenols can inhibit the NF-κB pathway, an inflammatory cascade that has been demonstrated to contribute to the progression of lipogenesis through the action of cytokines such as TNF-α [176,177]. This targeted modulation illustrates how polyphenols interact with metabolic pathways at multiple levels, thereby creating a cellular environment that is less susceptible to lipid accumulation and inflammatory responses [178,179].

Vitamins C and E exemplify the synergistic interaction between water- and fat-soluble antioxidants, with distinct solubility profiles that enable comprehensive cellular protection. Vitamin C’s role as a primary ROS neutralizer and vitamin E regenerator enhances its efficacy, particularly in adipocytes, where it facilitates the maintenance of vitamin E’s lipid-protective functions. Meanwhile, vitamin E, a lipid-soluble antioxidant, integrates into cellular membranes to prevent lipid peroxidation, a process that is often accelerated in obesity and linked to impaired membrane integrity and inflammatory signaling [128,148]. Collectively, these antioxidants facilitate redox homeostasis in adipose cells, thereby preventing ROS-driven inflammation and abnormal lipid deposition [180]. This cooperation demonstrates how different antioxidants can create a multi-faceted defense mechanism, effectively counteracting oxidative stress-induced lipogenesis and supporting cellular health [127,172].

The influence of natural antioxidants on metabolic health is substantiated by a substantial body of research indicating that diets rich in antioxidants are associated with reduced rates of obesity and improved metabolic profiles. The ingestion of polyphenol-rich foods, such as green tea and resveratrol, has been shown to result in a notable reduction in body fat and enhanced insulin sensitivity [181,182]. Recent evidence indicates that the combination of antioxidants, such as polyphenols with vitamin C, results in a synergistic effect that amplifies antioxidant capacity and further inhibits fat accumulation [183,184,185]. Table 2 summarizes the primary sources, roles, and mechanisms of dietary antioxidants in mitigating oxidative stress, highlighting their regulatory effects on lipogenesis and their positive impact on metabolic health.

Natural antioxidants protect against oxidative stress and help regulate lipids. This helps limit lipid accumulation and enhance metabolic resilience, making them important for preventing and managing obesity and metabolic disorders [209,210,211].

## 8. Therapeutic Potential of Antioxidants in Obesity and Metabolic Disorder Management

The role of antioxidants as a therapeutic tool in the management of obesity and associated metabolic disorders is becoming increasingly recognized. Dietary antioxidants, in particular, offer a non-invasive intervention that can combat the underlying oxidative stress and inflammation [212,213]. Diets rich in bioactive compounds, particularly the Mediterranean diet, have been demonstrated to be strongly associated with improvements in metabolic health, reduced rates of obesity, and enhanced insulin sensitivity [214,215,216]. For example, polyphenols present in olive oil and red wine act as direct scavengers of ROS while simultaneously promoting the activity of endogenous antioxidant enzymes, including GPx and SOD [217,218,219]. This action of neutralizing ROS restores redox balance in adipose tissue, thereby limiting inflammation and supporting normal lipid metabolism. These mechanisms enable dietary antioxidants to mitigate oxidative damage and inflammation in adipose tissue, which is otherwise susceptible to ROS-induced dysfunction that leads to insulin resistance and abnormal lipid storage [127,220,221]. The promotion of long-term metabolic stability and healthier fat tissue function is facilitated by the regulation of these pathways by antioxidant-rich diets.

In addition to the general advantages of dietary antioxidants, targeted antioxidant supplementation has been demonstrated to enhance cellular resilience to metabolic stress. However, it is essential to exercise caution when considering factors such as dosage and bioavailability. The efficacy of vitamins E and C, in conjunction with polyphenolic extracts derived from green tea and berries, has been demonstrated in the reduction of adipose tissue, improvement of glucose metabolism, and protection of adipose tissue from oxidative damage [104,105]. From a mechanistic standpoint, vitamin E serves to stabilize cell membranes by preventing lipid peroxidation, while vitamin C plays a role in regenerating vitamin E following oxidation, thereby extending its protective capabilities [93,128,222]. However, it should be noted that high-dose supplements may potentially interfere with physiological ROS signaling, which supports functions such as the immune response and insulin sensitivity [114]. Moreover, antioxidants impact metabolic regulation via the NF-κB pathway, which plays a pivotal role in inflammatory signaling and glucose metabolism [223,224]. By modulating NF-κB, antioxidants reduce cytokine release, improve insulin receptor activity, and enhance glucose uptake, which are all essential actions for the management of type 2 diabetes [80,189]. Excessive antioxidant intake may disrupt homeostatic mechanisms, necessitating careful balancing of benefits against individual metabolic contexts [225,226,227].

A personalized approach to antioxidant therapy represents a promising avenue for more precise treatments, given that individual variations in oxidative stress and metabolic responses influence therapeutic outcomes [228,229,230]. Given the sexually dimorphic nature of adipose tissue distribution, with women predominantly storing fat in subcutaneous depots and men in visceral regions, it is crucial to consider these differences when evaluating the potential role of antioxidants in metabolic interventions [231]. Antioxidant therapies may need to be personalized to address these sex-specific variations, with a focus on mitigating oxidative stress in subcutaneous adipose tissue for women and targeting inflammation and metabolic dysfunction in visceral fat for men [232,233]. Such tailored approaches could optimize therapeutic outcomes by aligning with the distinct metabolic challenges associated with each fat depot, ultimately contributing to more effective management of obesity and its related metabolic disorders.

Personalized antioxidant therapy is a therapeutic approach that utilizes biomarkers to guide intervention strategies. Biomarkers such as malondialdehyde (MDA), F2-isoprostanes, and 8-hydroxy-2′-deoxyguanosine (8-OHdG) serve as indicators of oxidative damage, while assays like total antioxidant capacity (TAC) and ferric reducing antioxidant power (FRAP) measure overall antioxidant potential [234,235,236]. Advances in genomics have led to the identification of genetic variations, including single-nucleotide polymorphisms (SNPs) in antioxidant defense genes, which influence individual responses to oxidative stress and antioxidant therapy. Integration of these tools enables the customization of interventions to account for genetic predispositions and metabolic conditions, thereby enhancing their efficacy [237,238,239]. The optimization of antioxidant intake through the consideration of oxidative stress markers, metabolic profiles, and genetic backgrounds can facilitate the realization of the benefits of antioxidants while mitigating the risks associated with over-supplementation [240,241,242]. For instance, individuals exhibiting elevated oxidative markers may derive benefit from a combination of antioxidants, such as polyphenols and vitamin C, which act in concert to enhance antioxidant capacity and target inflammation in adipose tissue [142,243]. Such personalization considers the inter-individual variability in the absorption, utilization, and response to antioxidants. As an emerging field in the study of metabolic health, personalized therapy also highlights the necessity of addressing the bioavailability challenges associated with many antioxidants, which often exhibit limited stability and absorption in supplement form. Novel delivery systems, including nanoencapsulation and liposomal formulations, are currently being investigated to enhance the stability, absorption, and efficacy of these compounds [244,245,246]. The potential to overcome these barriers to bioavailability could position antioxidants as central agents in the management of obesity and metabolic disorders, addressing both oxidative and inflammatory aspects of metabolic dysfunction in a patient-centered, targeted manner [247,248].

Although they show promising therapeutic potential, the role of antioxidants in adipogenesis and metabolic diseases remains contentious, as clinical trials frequently report that vitamin-based supplements fail to enhance health outcomes or extend longevity [249,250]. In certain instances, excessive antioxidant use has been observed to impede physiological ROS signaling, which is imperative for processes such as insulin sensitivity and immune regulation. These findings underscore the need for context-specific strategies to optimize antioxidant use [251].

## 9. Conclusions and Future Perspectives

This review highlights the critical function of antioxidants in addressing the pathophysiological processes underlying obesity and related metabolic disorders. Antioxidants, both endogenous and dietary, are indispensable for neutralizing ROS, reducing inflammation, and maintaining lipid and glucose homeostasis in adipose tissue. By upregulating antioxidant enzymes such as GPx and SOD, antioxidants assist in the mitigation of oxidative stress in adipocytes, thereby reducing lipid peroxidation and preserving cellular integrity. Moreover, antioxidants impact pivotal transcription factors, such as PPARγ and C/EBPα, which are vital regulators of adipogenesis, inflammation, and adipocyte hypertrophy—the primary drivers of obesity. These mechanisms collectively position antioxidants as a promising class of non-invasive therapeutic agents for the improvement of metabolic health, reduction of adipose tissue mass, and enhancement of insulin sensitivity.

Dietary antioxidants from fruits, vegetables, nuts, and whole grains—including polyphenols, flavonoids, carotenoids, and vitamins C/E—function as ROS scavengers while boosting endogenous antioxidant systems. For instance, berry anthocyanins and citrus-derived vitamin C neutralize ROS and regenerate oxidized antioxidants (e.g., recycling vitamin E), prolonging their efficacy. Similarly, polyphenols and vitamin E from greens and nuts inhibit lipid peroxidation, preserving adipocyte membrane integrity. Carotenoids further mitigate oxidative damage by quenching singlet oxygen. Collectively, these compounds reduce oxidative stress, improve lipid metabolism, enhance insulin sensitivity, and promote metabolic resilience.

Despite the promising effects of antioxidants, future research must refine their therapeutic use for obesity and metabolic disorders, with a particular emphasis on personalized interventions. In addition to the general benefits of antioxidant therapies, it is essential to recognize the sexually dimorphic nature of adipose tissue distribution when considering their application in metabolic interventions. This is due to the fact that while dysfunctional adipocytes are central to the development of obesity and metabolic-related diseases, strategies that indirectly target sexually dimorphic adipose tissue may also have beneficial outcomes. Personalizing antioxidant treatments in this way could optimize therapeutic outcomes, aligning interventions with the distinct metabolic challenges each sex faces. Ultimately, such tailored approaches hold significant promise for improving the management of obesity and associated metabolic disorders. It is important to note that individual responses to antioxidants may vary based on a number of factors, including genetic predispositions and oxidative stress levels. For example, single-nucleotide SNPs in antioxidant defense genes, such as glutathione S-transferase (GST), have been demonstrated to influence the body’s response to specific antioxidants. This indicates the possibility of personalized antioxidant therapies, in which metabolic profiles and oxidative stress markers could assist in optimizing antioxidant selection, dosage, and delivery while reducing the risk of over-supplementation. Furthermore, recent developments in antioxidant delivery systems, including nanoencapsulation and liposomal formulations, present promising strategies to overcome current limitations in bioavailability, stability, and absorption. Notably, research into the potential synergistic effects of combining antioxidants with other compounds, such as polyphenols with vitamins C and E, may reveal additive or even synergistic effects, offering a more comprehensive approach to protecting against metabolic dysfunction. Collectively, these advancements in personalized medicine and delivery technologies have the potential to fully unlock the therapeutic benefits of antioxidants, positioning them as critical components in the prevention and treatment of obesity and other metabolic disorders.

## Figures and Tables

**Table 1:** Roles and mechanisms of endogenous antioxidants in obesity-related oxidative stress.

Antioxidant	Primary Function in Response to Obesity-Related Oxidative Stress	Mechanism of Action	References
Glutathione (GSH)	It has been demonstrated that this compound is capable of neutralizing ROS and detoxifying lipid peroxides, thereby reducing oxidative damage to cell membranes and maintaining redox balance	Serves as an electron donor to convert ROS to less reactive molecules; recycles oxidized GSH with glutathione reductase	[156,157]
Superoxide Dismutase (SOD)	Converts superoxide radicals to hydrogen peroxide, protecting cellular components from ROS-induced damage, particularly in mitochondria	Catalyzes the dismutation of superoxide radicals; operates in mitochondria (Mn-SOD) and cytoplasm (Cu/Zn-SOD)	[127,158]
Catalase	Decomposes hydrogen peroxide into water and oxygen, preventing the accumulation of toxic ROS in adipose cells	Works in peroxisomes; breaks down hydrogen peroxide resulting from SOD activity	[98,159]
Glutathione Peroxidase (GPx)	Reduces hydrogen peroxide and organic peroxides, protecting lipids and cellular membranes from peroxidation	Uses GSH as a cofactor to convert lipid peroxides to non-toxic products; vital in lipid-rich environments	[156,160]
Thioredoxin System	Reduces oxidized protein thiols, maintaining protein stability and preventing cell damage from oxidative modifications	Involves thioredoxin reductase and thioredoxin to restore protein structure and function	[161,162]
Coenzyme Q10 (CoQ10)	Stabilizes the electron transport chain in mitochondria, reducing ROS production while supporting ATP synthesis	Transfers electrons in mitochondria, limiting ROS leakage during oxidative phosphorylation	[163,164]
Peroxiredoxins (PRXs)	Neutralizes hydrogen peroxide and organic hydroperoxides, providing additional protection against oxidative damage in adipose tissue	Catalyzes peroxide reduction using thioredoxin; involved in redox signaling and cellular stress response	[109]
Paraoxonase-1 (PON1)	Protects lipoproteins from oxidative modification, reducing lipid peroxidation and supporting vascular health in obesity	Hydrolyzes lipid peroxides in LDL and HDL, preventing foam cell formation and oxidative stress in the vascular system	[165,166]
Selenosugars	Reduce oxidative stress, particularly from lipid peroxidation in fat-rich environments like obesity. They protect cells and support tissue repair by counteracting ROS in high-stress conditions	Neutralize free radicals, especially lipid peroxides, by donating electrons; their phenolic structure enhances radical scavenging and boosts cellular antioxidant defenses, maintaining homeostasis in oxidative stress environments.	[167]
Heme Oxygenase-1 (HO-1)	Degrades heme into biliverdin, carbon monoxide, and iron, providing antioxidant effects and preventing heme-induced oxidative damage	Converts heme to biliverdin, which is further reduced to bilirubin, a potent antioxidant in cellular protection	[168,169]

**Table 2:** Roles and mechanisms of dietary antioxidants in obesity-related oxidative stress.

Antioxidant	Primary Sources	Role in Oxidative Stress	Mechanism in Lipogenesis Regulation	Impact on Metabolic Health	References
Tea Polyphenols	Green tea, grapes	Scavenge ROS, upregulate GPx and SOD	Downregulate PPARγ, C/EBPα;inhibit NF-κB, reducing cytokine release	Lowers fat accumulation; enhances antioxidant enzyme activity; reduces inflammation in adipose tissue	[186]
Vitamin C	Citrus fruits, bell peppers, greens	Neutralizes ROS, regenerates vitamin E	Lowers cytokine levels, reducing pro-lipogenic signals	Supports insulin sensitivity; reduces adipocyte inflammation; maintains vascular health	[187,188]
Vitamin E	Nuts, seeds, vegetable oils	Prevents lipid peroxidation, stabilizes cell membranes	Maintains adipocyte membrane integrity; prevents abnormal lipid storage	Reduces lipid oxidative damage; preserves membrane structure for metabolic stability	[92,93,128]
Flavonoids	Berries, onions, apples	Induce SOD and GPx, scavenge ROS	Suppress inflammatory cytokines; reduce adipocyte differentiation	Lowers inflammation, oxidative stress; supports lipid metabolism and metabolic flexibility	[133,189,190]
Carotenoids	Carrots, sweet potatoes, tomatoes	Quench singlet oxygen, reducing ROS	Stabilize membranes, limit adipocyte hypertrophy	Contributes to reduced adiposity; enhances antioxidant defenses, promotes lipid metabolism	[191,192]
Resveratrol	Red grapes, berries, peanuts	Activates SIRT1, reduces ROS	Downregulates PPARγ, limits adipocyte proliferation and lipid accumulation	Improves insulin sensitivity; lowers fat mass; promotes mitochondrial health	[193]
Curcumin	Turmeric	Inhibits ROS production, reduces lipid peroxidation	Modulates NF-κB pathway, lowering inflammation and lipogenesis	Reduces adipose tissue inflammation; improves lipid metabolism; aids in weight management	[194,195]
Quercetin	Apples, onions, capers	Stabilizes cell membranes, inhibits ROS production	Lowers PPARγ activity; limits cytokine production, reducing lipogenesis	Reduces adipocyte proliferation; lowers systemic inflammation; supports insulin function	[196,197]
Lycopene	Tomatoes, watermelon, pink grapefruit	Neutralizes ROS, protects lipids and DNA	Limits adipocyte size, promotes healthy lipid storage	Reduces metabolic syndrome risk; prevents lipid peroxidation; supports vascular health	[198,199]
Anthocyanins	Blueberries, cherries, blackcurrants	Scavenge ROS, protect mitochondrial function	Inhibit C/EBPα and NF-κB; reduce inflammation-driven lipogenesis	Lowers oxidative stress; decreases fat deposition; enhances insulin signaling	[200,201]
Silymarin	Milk thistle	Enhances SOD, GPx activity; stabilizes cell membranes	Suppresses NF-κB, reducing pro-inflammatory cytokines	Decreases lipid storage in liver; supports liver function; lowers oxidative stress	[202,203]
Bioactive Tamarind Compounds	Tamarind (*Tamarindus indica*)	Phenolic compounds scavenge ROS, reduce oxidative damage, and enhance antioxidant defenses.	Modulates pro-inflammatory pathways and downregulates lipogenic enzymes and cytokines, reducing lipid accumulation.	Supports cardiovascular and metabolic health by improving lipid metabolism, reducing systemic inflammation, and mitigating risks of chronic diseases (e.g., diabetes, cancer, heart disease).	[204,205,206]
*Syzygium cumini*	Java plum fruit and seeds	Rich in phenolic compounds, which can scavenge ROS and reduce oxidative damage	Regulates lipid metabolism by inhibiting adipocyte differentiation and reducing lipid accumulation	Reduces fat deposition, enhances antioxidant defenses, and supports cardiovascular and metabolic health	[207,208]
